# Supplementary material for: Detection of SARS-CoV-2 Delta Variant (B.1.617.2) in Domestic Dogs and Zoo Tigers in England and Jersey during 2021
Source: Viruses. 2024 Apr 16;16(4):617. doi: 10.3390/v16040617 (PMC11053977; doi:10.3390/v16040617)
Supplement: Supplementary file 1 [file viruses-16-00617-s001.zip › viruses-2920148-supplementary.pdf]

Supplementary Table S1. Assembled raw reads mapped to SARS-CoV-2/human/China/WIV04/2020 (GenBank Accession No: MN996528) as the reference sequence and deposited on NCBI Sequence Read Archive (SRA) under experiment accession number PRJNA1092720. The mapping statistics include the number of reads in the assembly, the percentage coverage of positions with at least one base aligned, the mean depth of coverage across the genome, the mean base quality score (Phred-scaled 0-40; the higher the quality score the more reliable the base call) and the mean mapping quality of the selected reads.

Sequence name	Accession numbers GISAID; GenBank; NCBI SRA	Number of reads	Coverage bases (%)	Mean depth of coverage	Mean base quality	Mean mapping quality
SARS-CoV-2/dog/Jersey/M8-21-02/2021	EPI_ISL_18943724; PP515674; SRX24081943	53687	29714 (99.41%)	249.746	35.2	59.9
SARS-CoV-2/dog/England/M11-21-01/2021	EPI_ISL_18943725; PP515675; SRX24081944	54654	29842 (99.84%)	258.652	38.4	60.0
SARS-CoV-2/tiger/England/M12-21-01/2021	EPI_ISL_18943726; PP515676; SRX24081945	58115	29866 (99.92%)	276.85	34.6	59.7
SARS-CoV-2/tiger/England/M12-21-05/2021	EPI_ISL_18943727; PP515677; SRX24081946	11677	29129 (97.45%)	36.6328	28.3	55.1
SARS-CoV-2/tiger/England/M12-21-07/2021	EPI_ISL_18943728; PP515678; SRX24081947	53543	29667 (99.25%)	251.423	34.4	59.6
SARS-CoV-2/dog/England/M14-21-11/2021	EPI_ISL_18943729; PP515679; SRX24081948	49844	29493 (98.67%)	229.908	33.8	59.7
